# The Role of Psychosomatic Traits in Tailored Workup for Anterior Cervical Discectomy and Fusion—A Case Series

**DOI:** 10.3390/jpm14050454

**Published:** 2024-04-25

**Authors:** Marco Battistelli, Edoardo Mazzucchi, Mario Muselli, Filippo Maria Polli, Gianluca Galieri, Paola Bazzu, Fabrizio Pignotti, Alessandro Olivi, Giovanni Sabatino, Giuseppe La Rocca

**Affiliations:** 1 Department of Neuroscience, Neurosurgery Section, Università Cattolica del Sacro Cuore, 00168 Rome, Italy; gianluca.galieri01@icatt.it; 2 Department of Neurosurgery, Mater Olbia Hospital, 07026 Olbia, Italy; edoardo.mazzucchi@materolbia.com (E.M.); fabrizio.pignotti@materolbia.com (F.P.); 3 Department of Life, Health and Environmental Sciences, University of L’Aquila, 67100 L’Aquila, Italy; mario.muselli@univaq.it; 4 Department of Neurosurgery, Fondazione Policlinico Universitario A. Gemelli IRCCS, 00168 Rome, Italy; filippomaria.polli@policlinicogemelli.it (F.M.P.); alessandro.olivi@policlinicogemelli.it (A.O.); giovanni.sabatino@policlinicogemelli.it (G.S.); giuseppe.larocca@policlinicogemelli.it (G.L.R.); 5 Psychology and Psychotherapy Service, Mater Olbia Hospital, 07026 Olbia, Italy; paola.bazzu@materolbia.com

**Keywords:** case-report, cervical spine degenerative, ACDF, SCL-90, psychosomatic traits, PROMs, psychology, outcome

## Abstract

Study design: Prospective study. Objective: To evaluate the influence of preoperatively assessed psychosomatic traits on postoperative pain, disability, and quality of life outcomes. Summary of background data: Anterior cervical discectomy and fusion (ACDF) is a widely employed surgical procedure for treating cervical spondylosis. Despite its effectiveness, various studies have reported non-success rates in terms of alleviating disability and pain. Psychological factors have become increasingly recognized as critical determinants of surgical outcomes in various medical disciplines. The full extent of their impact within the context of ACDF remains insufficiently explored. This case series aims to assess the influence of preoperative psychological profiling on long-term pain, disability and quality of life outcomes. Methods: We conducted a prospective cohort study of prospectively collected data from 76 consecutive patients who underwent ACDF with PEEK inter-fixed cages from July 2019 to November 2021. The preoperative psychological traits were assessed using the Symptom Checklist 90 (SCL-90) questionnaire. The Oswestry Disability Index (ODI), Visual Analogue Scale (VAS), Neck Disability index (NDI), EuroQol-5D (EQ-5D), and Short Form-36 (SF-36) were collected preoperatively, one month postoperatively, and at least one year after the surgical procedure. Results: The correlation analyses revealed associations between psychosomatic traits and multiple preoperative and postoperative outcome measures. The univariate analyses and linear regression analyses demonstrated the influence of the Global Severity Index (GSI) over the final follow-up scores for the ODI, VAS, NDI, EQ-5D, and SF-36. The GSI consistently exhibited a stronger correlation with the final follow-up pain, disability, and quality of life outcomes with respect to the correspondent preoperative values. Conclusion: This study highlights the importance of psychosomatic traits as predictive factors for ACDF outcomes and emphasizes their relevance in preoperative assessment for informing patients about realistic expectations. The findings underscore the need to consider psychological profiles in the preoperative workup, opening avenues for research into medications and psychological therapies. Recognizing the influence of psychosocial elements informs treatment strategies, fostering tailored surgical approaches and patient care.

## 1. Background

Anterior cervical discectomy and fusion (ACDF) is a cornerstone procedure for managing degenerative and traumatic cervical spine conditions [1]. Several factors have been shown to be predictors for ACDF success in treating myelopathy or radiculopathy, such as smoking habits [2], postoperative dysphagia [3], preoperative disability and pain level, gender, cervical spine sagittal profile [4], cage design and material, subsidence, pseudoarthrosis [5], and the active range of motion (ROM) [6]. Psychological elements, like maladaptive coping strategies and psychiatric comorbidity, may contribute to the persistence of pain and have often been overlooked. Recently, there has been growing interest in assessing the preoperative psychological status as it is recognized to affect surgical outcomes. For instance, preoperative anxiety and depression have been recognized to relate to an increased likelihood of postoperative complications, extended length of hospital stay, and/or hospital readmission in females across various surgical disciplines, such as cardiac, colon–rectum, ear–nose–throat, general, gynecology, orthopedic, plastic, urogynecology, urology, and vascular surgery [7]. Anxiety and depression have also been linked to lower quality of life (QoL) and increased postoperative pain scores in a study of 133 patients who underwent surgical resection for pulmonary ground-glass opacities [8]. Consequently, spine surgeons are increasingly exploring how specific psychological profiles might influence postoperative surgical outcomes. Targeted studies have been conducted across various spine disorders, including failed back surgery syndrome (FBSS) [9], lumbar disc herniation [10], lumbar arthrodesis [11], and lumbar decompression [12]. The present case series aims to fill the gap in the available literature concerning the impact of psychopathologic traits on the surgical outcome of ACDF and the role of psychological profiling in tailoring surgical planning.

The Symptom Checklist 90 (SCL-90) is a widely utilized questionnaire for assessing symptomatic distress, demonstrating adequate measurement consistency. It has proven to be a valuable tool for evaluating overall psychopathology [13]. One of its primary advantages lies in its capacity to stratify patients into nine distinct psychological profiles, obviating the need for direct involvement from surgeons or psychologists, as it can be administered directly to patients.

This study aims at evaluating the impact of preoperative psychological profiles, as assessed by the SCL-90 questionnaire, on both short-term and long-term outcomes following ACDF. Specifically, our focus is on assessing their influence on preoperative and postoperative pain levels, disability, and quality of life. These findings hold implications for the preoperative counseling of patients, as well as for further research exploring the potential benefits of preoperative interventions, such as medication or psychological counseling, when certain psychopathological profiles are identified. Our ultimate message is to emphasize the importance of understanding and addressing the preoperative psychological state of ACDF patients to optimize postoperative outcomes and promote future investigations in this critical area.

## 2. Methods

### 2.1. Patient Selection

We conducted a prospective study on a cohort of patients who underwent ACDF with the implantation of PEEK cages (CoRoent^®^ Small InterlockTM system, NuVasive^®^, San Diego, CA 92121, USA) for degenerative cervical diseases between July 2019 and November 2021 at the teaching Neurosurgery Department of Mater Olbia Hospital—Catholic University of the Sacred Heart in Italy. Inclusion criteria were ACDF on a level between C3 and C7, patient’s consent to undergo preoperative assessment of psychopathological profile through SCL-90 and PROM questionnaires (SF-36, VAS, OSI, NDI, EQ-5D), and willingness to fulfill PROM questionnaires at regular follow-ups at one month and at least one year after the surgical procedure. Exclusion criteria were less than one year FU, prior cervical procedures, level of surgery other then C3-C7, or unwillingness to undergo follow-up questionnaires.

### 2.2. Clinical Assessment

We assessed multiple parameters at three distinct time points: preoperatively (within one month prior to the surgery), one month postoperatively, and at the final follow-up (fFU). These parameters included the Visual Analogue Scale (VAS), the Short Form-36 Health Survey physical score and mental score (SF-36(PS) and SF-36(MS)), the Neck Disability Index (NDI), the Oswestry Disability Index (ODI), and the EuroQol-5D (EQ-5D).

### 2.3. Psychological Profile Assessment and Psychological Distress Test

The Italian version of the SCL-90 questionnaire was administered to patients preoperatively, within one month prior to their surgery. The Italian version of SCL-90 has undergone validation and exhibits strong internal consistency across all subscales (α values ranging from 0.70 to 0.96) [14]. The SCL-90 serves as a comprehensive psychological screening tool evaluating various dimensions of psychological distress. Participants are asked to rate the severity of 90 distress-related symptoms on a 5-point Likert scale, with 0 indicating ‘not at all’ and 4 indicating ‘extremely.’ Patients are instructed to indicate how bothered they were by each symptom over the previous week. These statements are categorized into nine distinct dimensions or factors (F), each reflecting different aspects of psychopathology: (F1) somatization, (F2) obsessive compulsive tendencies, (F3) interpersonal sensitivity, (F4) depression, (F5) anxiety, (F6) hostility, (F7) phobic anxiety, (F8) paranoid ideation, and (F9) psychoticism. In addition to these dimensions, three supplementary global indices quantify the degree of symptomatology: the Global Severity Index (GSI) measures the overall severity of all answered statements by calculating the average depth of impairment; the Positive Symptom Total index (PST) counts the total number of symptoms experienced; the Positive Symptom Distress Index (PSDI) evaluates the level of distress while accounting for the number of reported symptoms [15]. Of the three global indices, the GSI is the most sensitive indicator of the respondent’s distress level and combines information about the number of symptoms and the intensity of distress. It is calculated using the sum of the nine symptom dimensions and dividing by the total number of items to which the individual responded.

### 2.4. Statistical Analyses

After data normality assessment using the Shapiro–Wilk test, comparisons between baseline and follow-up of continuous variables were performed using Wilcoxon signed-rank test. Univariate linear regression was used to investigate the association between preoperative GSI and its subscales and ODI, VAS, NDI, SF-36, and EQ-5D scores at follow-up. The multivariable regression model was used to determine the association of various risk factors on ODI at fFU. Spearman’s rank correlation was used to assess the relationship between continuous variables. For each analysis, an alpha level of 0.05 was considered to be statistically significant. The statistical analysis was performed using the STATA 17 (StataCorp LLC, 4905, Lakeway Drive, College Station, TX, USA) software for Windows for all data analyses.

### 2.5. Ethical Approval

All patients signed a written informed consent form. The study was previously approved by the local ethics committee, protocol number 276/2020/CE. This case series has been reported in line with the Preferred Reporting of Case Series in Surgery (PROCESS) guidelines [16].

## 3. Results

Seventy-six consecutive patients, consisting of 44 females (57.9%) and 32 males (42.1%), were included in the study. At baseline, the mean age was 51.8 ± 10.0 years, ranging from 34 to 75 years (Table 1); the patients had a mean VAS score of 7.3, a mean ODI score of 43%, and a mean GSI score of 0.61. As shown in Table 2, there was a significant decrease in the average VAS, NDI, and ODI score between the baseline and fFU values (*p* < 0.05). Instead, a statistically significant increase was observed in the mean scores of the SF-36 (45.2 vs. 58.3; *p* = 0.0008) and EQ-D5 (0.5 vs. 0.7; *p* < 0.0001).

The univariate linear regression analyses showed the association between the preoperative psychopathological traits (somatization, obsessive compulsive tendencies, interpersonal sensitivity, depression, anxiety, hostility, phobic anxiety, paranoid ideation, psychoticism, and GSI), and the fFU VAS, NDI, ODI, SF-36, and EQ-5D scores, as summarized in Table 3. The baseline GSI score exhibited a significant positive association with the ODI, VAS, and NDI, and a negative correlation with the SF-36 and EQ-5D (*p* < 0.05). Similarly, the somatization, obsessive compulsive tendencies, depression, and anxiety subscales also had a significant positive association with the ODI, VAS, and NDI and a negative association with the SF-35 and ED-Q5.

The phobic anxiety and paranoid ideation subscales showed a significant positive association only with the NDI. The hostility subscale showed a significant positive association with the NDI and a negative association with the SF-35; the psychoticism subscale had a significant positive association with the ODI and NDI and a negative association with the SF-35 and ED-Q5. Finally, the interpersonal sensitivity subscale showed no association with any of the scores considered.

Spearman’s rank correlation highlighted multiple associations between the preoperative psychosomatic traits and the preoperative and fFU VAS, NDI, ODI, SF-36, and EQ-5D scores (Supplementary Table S1).

Additionally, we have constructed multiple linear regression models for each of the values for the follow-ups of the ODI, VAS, NDI, SF-36, and EQ-D5, considering the GSI and its respective preoperative value as possible predictors (Table 4). Notably, the preoperative GSI was the only significant predictor of the ODI, VAS, and SF-36 fFU scores. Instead, for the fFU scores of the NDI and EQ-D5, both the GSI and its respective preoperative scores were statistically significant predictors.

## 4. Discussion

ACDF is indeed a highly effective surgical procedure for treating several cervical diseases such as spondylosis and trauma. The procedure bears a low risk of complications and side effects, allowing patients to regain their normal daily life activities, including participation in professional sports [17,18]. One of the major healthcare issues in recent years consists of reducing costs while maintaining optimal surgical results. In this view, tailoring surgical care to patients’ characteristics can prove effective in reducing health-related costs, on top of improving patients’ satisfaction. Therefore, it becomes increasingly important to preoperatively assess potential risk factors that may predict unfavorable outcomes. With that in mind, we stress the importance of incorporating the psychopathological profiling of patients into the workup for ACDF planning. This study reveals the significant influence of each preoperative psychopathological trait, namely, somatization, obsessive compulsive tendencies, interpersonal sensitivity, depression, anxiety, hostility, phobic anxiety, paranoid ideation, and psychoticism, on long-term pain, disability, and quality of life parameters. Moreover, the higher influence of preoperative psychological global distress, as assessed by GSI, over long-term pain, disability and quality-of-life parameters emerges with respect to their correspondent severity.

Numerous studies in the literature have sought to report risk factors of ACDF failure in terms of alleviating disability, arm pain, and neck pain. For instance, a case series by C. Mjåset et al., conducted using data from the Norwegian Registry for Spine Surgery (NORspine), revealed non-success rates of 38.0% for neck disability and 35.3% for arm pain following ACDF procedures performed for cervical degenerative radiculopathy. Non-success was defined as NDI > 26 and/or an arm numerical rating scale (NRS) > 2 at the 12-month follow-up. The predictors for non-success in terms of neck disability included engaging in physically demanding work, lower levels of education, pending litigations, previous neck surgery, arm pain duration exceeding three months, and medium-to-high levels of baseline disability, along with anxiety and depression [19]. The predictors for non-success in terms of arm pain included having a non-Norwegian mother tongue, smoking, medium-to-high levels of baseline arm pain, and all neck disability model predictors except for anxiety and depression [17]. While this pivotal study included an evaluation of the psychopathological traits in the context of factors which affect the ACDF outcome, it appeared to be limited by the employment of the EQ-5D questionnaire, which is not a structured questionnaire specifically aimed at addressing the psychopathological profile. In the modern era of surgical practice, it is increasingly recognized that, in addition to pain and disability, psychosocial factors are fundamental in patients’ recovery and must be taken into account when selecting an appropriate treatment. A retrospective cohort study conducted by R.A. LaCaille et al. employed a multivariable model to predict factors that influence outcomes on seventy-three patients who underwent lumbar interbody fusion. Their findings revealed a statistically significant association between depression and various aspects of physical disability related to lumbar back pain, as assessed by the Roland–Morris Disability Questionnaire (RDQ), as well as the quality of life, as assessed using the SF-36 questionnaire. Additionally, smoking habits and pending litigations were found to be linked with poorer disability and quality of life outcomes [20]. It is worth noting that the diagnosis of depression in this study was based on patients’ clinical histories, specifically as one of the biopsychosocial factors that could impact prognosis. The approach used by the authors to assess the patients’ psychopathological status was mainly biased by its dichotomic nature, since patients were stratified based on the diagnosis of this psychiatric disorder, while untreated depressive psychosomatic traits may have been included in the treatment as well as in the control group. The degree of depression correction with appropriate medications was not taken into account in assessing the pain and quality of life outcomes. Moreover, several other psychological disorders were not considered. R.J. Marek et al. conducted a retrospective review of 603 patients who sought treatment at the Texas Back Institute and underwent various spinal procedures, including spine fusion, artificial disc replacement, laminectomy/discectomy/decompression, hardware removal, discography, or rhizotomy. To assess the psychological profiles, they employed the Minnesota Multiphasic Personality Inventory-2-Reconstructed Form (MMPI-2-RF). Their analysis revealed correlations between several MMPI-2-RF substantive scales and postoperative outcomes, including pain levels, Oswestry Disability Index (ODI) scores, negative affect, and patient dissatisfaction with surgical results. Specifically, factors such as demoralization, somatic complaints, low positive emotions, dysfunctional negative emotions, malaise, suicidal ideation, inefficacy/self-doubt, anger proneness, family problems, social avoidance, and negative emotionality were associated with poorer postoperative outcomes [21]. It is worth noting that a prior pilot study conducted by the same group of authors yielded similar findings, reinforcing the importance of considering psychological factors in the context of spine surgery and their potential impact on patient outcomes [22]. However, those studies were limited by heterogeneity in the inclusion criteria, since the patients included underwent different procedures, which may have biased the results.

In our study, we utilized the SCL-90 questionnaire to comprehensively characterize psychosomatic traits, including somatization, obsessive compulsive tendencies, interpersonal sensitivity, depression, anxiety, hostility, phobic anxiety, paranoid ideation, and psychoticism, on a uniform sample of patients who underwent ACDF performed with zero-profile PEEK cages. One of the SCL90’s main advantages relies on its ability to grade psychopathological profiles as continuous variables. This allowed us to evaluate the postoperative functional and quality of life results with respect to a full spectrum of the severity of a certain trait. Moreover, its comprehensive nature and the numerous amounts of questions for each of the traits permitted us to completely assess the patients’ psychological profile and to detect even minimal difference in the grading of a certain trait between each of the participants. Univariate analyses were used to explore the relationship between each psychological trait and surgical outcomes in terms of pain, disability, and quality of life. As indicated in Table 3, we were able to establish an association between higher Global Severity Index (GSI) scores and worse outcomes across all the assessed parameters. Of the collected psychosomatic traits, only interpersonal sensitivity did not appear to be linked to any of the outcomes. Phobic anxiety and paranoid ideation were associated solely with worse NDI scores at the fFU. The psychological traits that consistently demonstrated an adverse impact on outcomes across all the domains were somatization, obsessive compulsive tendencies, depression, and anxiety. These findings align with prior research, although it is important to note that none of the previous studies specifically focused on the treatment of cervical degenerative diseases, as we have in our investigation.

In the retrospective review from Marek et al. [21] it is worth highlighting that several of the analyzed psychological profiles also exhibited associations with pre-surgical pain levels, ODI scores, and negative affect, albeit with a weaker correlation. This observation raises the concern that, to some extent, preoperative pain, decreased quality of life, and disability might be attributed to psychosomatic dysfunctional traits. In our study, the correlation analyses yielded results similar to those observed in the study conducted by R.J. Marek et al. (Supplementary Table S1). However, the multiple linear regression analysis unveiled a critical distinction: the preoperative GSI showed a significant influence over the fFU ODI, VAS, and SF-36, while the correspondent preoperative ODI, VAS, and SF-36 did not (Table 4). Even when the preoperative values demonstrated a statistically significant influence on the fFU outcomes, as observed in the cases of the Neck Disability Index (NDI) and EuroQol-5D (EQ-5D), the preoperative GSI consistently exhibited a stronger correlation with the fFU values (Table 4). These results underscore the major relevance of preoperative global psychological distress on the final long-term results. To our knowledge, this is the first case series to report similar results and place emphasis on the importance of considering patients’ psychological profiling workup in the process of weighting the expected risks and supposed benefits of an ACDF procedure for a certain patient. However, we did not perform any interventions aimed at treating the psychopathological traits detected through the SCL-90 test. Future studies in this field should assess the ACDF outcomes in patients who are treated pharmacologically and/or with psychological therapies for their psychopathologic traits.

### Limitations

The present case series is firstly limited by its design, given its retrospective nature. The monocentric nature of this study could be considered a limitation of the generalizability of the results, but it is also, in our opinion, a strength of the present study, limiting the source of bias due to several surgeons using variable surgical techniques in different contexts of care. The sample size represents another possible limitation, increasing the risk of selection biases and reducing the results’ generalizability. While it was out of the scope of the present paper, future studies should consider the potential confounding factors that are reported to significantly impact ACDF outcomes to assess the weight of psychological traits in a comprehensive evaluation.

## 5. Conclusions

In conclusion, this study underscores the significance of considering psychological factors in the context of anterior cervical discectomy and fusion (ACDF) for cervical spondylosis, providing valuable insights for the neurosurgical community. It highlights the necessity of preoperative psychological assessments in guiding patient counseling and treatment decisions, recognizing the substantial impact of psychosomatic traits, particularly those measured by the Global Severity Index (GSI), on postoperative outcomes. As we move forward, the findings of this research unveil a critical dimension of ACDF success, prompting future studies to delve deeper into the intricate relationship between psychological profiles and treatment outcomes. Particularly, investigations into the potential benefits of preoperative medications or psychological therapies are of significant interest. Recognizing the role of psychosocial elements not only enhances patient well-being but also paves the way for refined surgical approaches, optimized patient care, and a more comprehensive understanding of cervical spine spondylosis treatment.

## Figures and Tables

**Table 1:** Sample characteristics.

Variable	Mean ± SD or n (%)
Age	51.8 ± 10
Male/Female	32 (42.1%)/44 (57.9%)
BMI	25.5 ± 4.4
Preop-ODI	0.4 ± 0.2
Preop-VAS	7.3 ± 1.8
Preop-NDI	0.6 ± 0.2
Preop-SF-36	45.2 ± 19.2
Preop-EQ-5D	0.5 ± 0.2
Preop-GSI	0.6 ± 0.6
Somatization	1.3 ± 0.8
Obsessive compulsive tendencies	0.9 ± 0.8
Interpersonal sensitivity	0.4 ± 0.5
Depression	0.7 ± 0.7
Anxiety	0.6 ± 0.7
Hostility	0.4 ± 0.6
Phobic anxiety	0.4 ± 0.7
Paranoid ideation	0.5 ± 0.6
Psychoticism	0.3 ± 0.5

Preop = preoperative.

**Table 2:** Baseline and follow-up ODI, NDI, VAS, SF-36, EQ-5D comparison.

Variable	Baseline	Follow-Up	*p*-Value
ODI	0.4 ± 0.2	0.2 ± 0.2	*p* < 0.001
VAS	7.3 ± 1.8	3.6 ± 2.8	*p* < 0.001
NDI	0.6 ± 0.2	0.3 ± 0.2	*p* < 0.001
SF-36	45.2 ± 19.2	58.3 ± 23.9	0.0008
EQ-5D	0.5 ± 0.2	0.7 ± 0.2	*p* < 0.001

*p* < 0.05 was considered statistically significant.

**Table 3:** Univariate analyses between ODI, VAS, NDI, SF-36, and EQ-5D fFU scores and SCL-90 subscales.

	ODI	VAS	NDI	SF-36	EQ-D5
GSI	0.2 (**<0.0001**)	2.18 (**0.011**)	0.27 (**<0.0001**)	−24.72 (**<0.0001**)	−0.22 (**0.003**)
Somatization	0.14 (**<0.0001**)	1.66 (**0.001**)	0.19 (**<0.0001**)	−18.26 (**<0.0001**)	−0.14 (**<0.0001**)
Obsessive compulsive tendencies	0.1 (**0.002**)	0.99 (**0.047**)	0.14 (**<0.0001**)	−12.72 (**0.001**)	−0.13 (**0.003**)
Interpersonal sensitivity	0.09 (0.142)	0.37 (0.696)	0.1 (0.190)	−11.23 (0.144)	−0.09 (0.274)
Depression	0.15 (**<0.0001**)	1.71 (**0.005**)	0.19 (**<0.0001**)	−18.07 (**<0.0001**)	−0.15 (**0.005**)
Anxiety	0.17 (**<0.0001**)	2.07 (**0.001**)	0.22 (**<0.0001**)	−18.47 (**<0.0001**)	−0.18 (**0.001**)
Hostility	0.1 (0.063)	1.31 (0.108)	0.15 (**0.022**)	−14.59 (**0.028**)	−0.11 (0.110)
Phobic anxiety	0.12 (0.131)	1.41 (0.287)	0.2 (**0.045**)	−17.53 (0.078)	−0.12 (0.298)
Paranoid ideation	0.09 (0.124)	0.99 (0.278)	0.17 (**0.019**)	−11.68 (0.118)	−0.12 (0.101)
Psychoticism	0.24 (**0.001**)	2.18 (0.053)	0.26 (**0.004**)	−22.81 (**0.013**)	−0.22 (**0.023**)

*p*-value < 0.05 was considered statistically significant.

**Table 4:** Multiple linear regression analysis.

**Dep.Var. FU-ODI**	**Coef.**	**95% CI**	***p*-value**
Preop-ODI	0.16	−0.08–0.39	0.186
GSI	0.19	0.09–0.3	0.001
**Dep.Var. FU-VAS**	**Coef.**	**95% CI**	***p*** **-value**
Preop-VAS	0.33	−0.07–0.72	0.101
GSI	1.89	0.23–3.56	0.027
**Dep.Var. FU-NDI**	**Coef.**	**95% CI**	***p*** **-value**
Preop-NDI.	0.4	0.05–0.76	0.028
GSI	0.2	0.07–0.34	0.004
**Dep.Var. FU-SF-36**	**Coef.**	**95% CI**	***p*** **-value**
Preop-SF-36	0.26	−0.05–0.56	0.097
GSI	−21.55	−34.73–−8.37	0.002
**Dep.Var. FU-EQ-5D**	**Coef.**	**95% CI**	***p*** **-value**
Preop-EQ-5D	0.33	0.06–0.61	0.019
GSI	−0.19	−0.32–−0.05	0.007

*p* < 0.05 was considered statistically significant; Dep. Var. = dependent variable; FU = follow-up.

## Data Availability

All data are in possess of senior author and can be requested for visualization.

## Supplementary Materials

The following supporting information can be downloaded at https://www.mdpi.com/article/10.3390/jpm14050454/s1, Table S1: Correlation analyses between psychosomatic profiles and preoperative and postoperative pain, disability and quality-of-life parameters.
