# Supplementary material for: The Role of Psychosomatic Traits in Tailored Workup for Anterior Cervical Discectomy and Fusion—A Case Series
Source: J Pers Med. 2024 Apr 25;14(5):454. doi: 10.3390/jpm14050454 (PMC11122414; doi:10.3390/jpm14050454)
Supplement: Supplementary file 1 [file jpm-14-00454-s001.zip › jpm-2960393-supplementary.pdf]

**Supplementary Table S1.** Correlation analyses between psychosomatic profiles and preoperative and postoperative pain, disability and quality-of-life parameters.

| | | Somatization | Obsessive-compulsive | Interpersonal sensitivity | Depression | Anxiety | Hostility | Phobic anxiety | Paranoid ideation | Psychoticism |
|---|---|---|---|---|---|---|---|---|---|---|
| **Preop-ODI** | Rho | 0.241 | 0.255 | 0.17 | 0.289 | 0.237 | 0.203 | 0.201 | 0.123 | 0.111 |
| | p-value | 0.05 | 0.037 | 0.169 | 0.018 | 0.053 | 0.1 | 0.102 | 0.321 | 0.37 |
| **FinalFU-ODI** | Rho | 0.546 | 0.403 | 0.197 | 0.482 | 0.518 | 0.247 | 0.202 | 0.206 | 0.432 |
| | p-value | < .001 | 0.002 | 0.142 | < .001 | < .001 | 0.063 | 0.131 | 0.124 | < .001 |
| **Preop-VAS** | Rho | 0.326 | 0.31 | 0.135 | 0.306 | 0.296 | 0.279 | 0.255 | 0.169 | 0.088 |
| | p-value | 0.007 | 0.011 | 0.277 | 0.012 | 0.015 | 0.022 | 0.038 | 0.173 | 0.479 |
| **FinalFU-VAS** | Rho | 0.446 | 0.269 | 0.054 | 0.376 | 0.429 | 0.219 | 0.146 | 0.149 | 0.263 |
| | p-value | < .001 | 0.047 | 0.696 | 0.005 | 0.001 | 0.108 | 0.287 | 0.278 | 0.053 |
| **Prop-NDI** | Rho | 0.478 | 0.467 | 0.341 | 0.519 | 0.453 | 0.383 | 0.355 | 0.341 | 0.247 |
| | p-value | < .001 | < .001 | 0.005 | < .001 | < .001 | 0.001 | 0.003 | 0.005 | 0.044 |
| **FinalFU-NDI** | Rho | 0.62 | 0.461 | 0.179 | 0.522 | 0.557 | 0.308 | 0.271 | 0.315 | 0.383 |
| | p-value | < .001 | < .001 | 0.19 | < .001 | < .001 | 0.022 | 0.045 | 0.019 | 0.004 |
| **Preop-SF-36** | Rho | -0.44 | -0.383 | -0.228 | -0.407 | -0.363 | -0.276 | -0.235 | -0.183 | -0.237 |
| | p-value | < .001 | 0.001 | 0.063 | < .001 | 0.003 | 0.024 | 0.055 | 0.138 | 0.053 |
| **FinalFU-SF-36** | Rho | -0.595 | -0.412 | -0.196 | -0.476 | -0.461 | -0.292 | -0.235 | -0.209 | -0.328 |
| | p-value | < .001 | 0.001 | 0.144 | < .001 | < .001 | 0.028 | 0.078 | 0.118 | 0.013 |
| **Preop-EQ-5D** | Rho | -0.33 | -0.332 | -0.326 | -0.401 | -0.357 | -0.321 | -0.379 | -0.308 | -0.272 |
| | p-value | 0.006 | 0.006 | 0.007 | < .001 | 0.003 | 0.008 | 0.002 | 0.011 | 0.026 |
| **FinalFU-EQ-5D** | Rho | -0.47 | -0.391 | -0.149 | -0.37 | -0.43 | -0.216 | -0.141 | -0.222 | -0.304 |
| | p-value | < .001 | 0.003 | 0.274 | 0.005 | < .001 | 0.11 | 0.298 | 0.101 | 0.023 |
